# Predictors of Post‐Bariatric Surgery Hypoglycemia: A Multicenter Retrospective Cohort Study

**DOI:** 10.1002/edm2.70123

**Published:** 2025-11-09

**Authors:** Mansour Bahardoust, Ali Ranjbarpazuki, Mohammadsadra Shamohammadi, Alla Mousavi, Danyal Yarahmadi, Meisam Haghmoradi, Babak Goodarzy, Armaghan Abbasi Garavand, Adnan Tizmaghz

**Affiliations:** ^1^ Department of Epidemiology, School of Public Health Shahid Beheshti University of Medical Sciences Tehran Iran; ^2^ School of Medicine Iran University of Medical Sciences Tehran Iran; ^3^ Gastrointestinal and Liver Diseases Research Center Iran University of Medical Sciences Tehran Iran; ^4^ Department of Orthopaedic Surgery Urmia University of Medical Sciences Urmia Iran; ^5^ Firoozabadi Clinical Research Development Unit (F A CRD U) is located at the Iran University of Medical Sciences (IUMS) in Tehran Tehran Iran

**Keywords:** bariatric surgery, post‐bariatric hypoglycemia, RYGB, sleeve gastrectomy

## Abstract

**Introduction:**

Post‐bariatric hypoglycemia (PBH) is a recognized complication that typically occurs within 1–3 years after bariatric surgery. We aimed to identify predictors of PBH in a large multicenter cohort.

**Materials and Methods:**

This retrospective, multicenter cohort study reviewed the medical records of 952 obese patients (body mass index (BMI) ≥ 35 kg/m^2^) who underwent Roux‐en‐Y gastric bypass (RYGB) or sleeve gastrectomy (SG) between 2020 and 2024 at three medical centers. PBH in a patient after bariatric surgery was defined as biochemically confirmed hypoglycemia less than 3.0 mmol/L (54 mg/dL) with typical symptoms of hypoglycemia, according to Whipple's triad. PBH incidence was evaluated at 12, 15, and 18 months postoperatively. Multivariable logistic regression was used to estimate adjusted associations with PBH after 12 months.

**Results:**

Cumulative PBH incidence was 25.9% at 12 months, 29.3% at 15 months, and 35.4% at 18 months. Factors associated with increased PBH risk included female sex (Odds Ratio (OR): 1.91, 95% Confidence Interval: 1.11–2.71), high school graduate (OR: 1.61, 95% CI: 1.10–2.11), vitamin B1/B12 deficiency (OR: 1.45, 95% CI: 1.04–1.85), and RYGB surgery (OR: 1.81, 95% CI: 1.11–2.51). Protective factors included having type 2 diabetes (OR: 0.75, 95% CI: 0.55–0.96), higher baseline HbA1c (OR: 0.97, 95% CI: 0.95–0.99), and longer diabetes duration (OR: 0.95, 95% CI: 0.91–0.99).

**Conclusion:**

PBH is a challenging complication after bariatric surgery. Our findings underscore the importance of considering metabolic, sociodemographic, and nutritional factors in assessing the risk of PBH.

## Graphical abstract

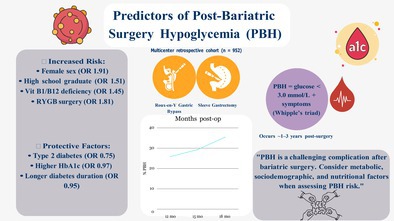

**Abbreviations**
BMI: body mass index
CGM: continuous glucose monitoring
CI: confidence Interval
FBS: fasting blood sugar
HER: electronic health record
OGTT: oral glucose tolerance test
OR: odds ratio
PBH: post‐bariatric hypoglycemia
RYGB: Roux‐en‐Y Gastric Bypass
SG: sleeve gastrectomy

## 1 Introduction

Obesity is a major global health concern that is associated with an increased risk of physical and psychological disorders [1]. Bariatric surgery has been established as a safe and effective therapeutic intervention for morbid obesity that achieves substantial and sustained weight loss, induces remission of diabetes and hypertension, and improves obesity‐related comorbidities [2, 3]. Globally, sleeve gastrectomy (SG) is the most common primary bariatric procedure, while Roux‐en‐Y gastric bypass (RYGB) is the most common revisional operation [4].

Post‐bariatric hypoglycemia (PBH) is a recognised complication following bariatric surgery that typically occurs within 1–3 years after the procedure [3, 5]. PBH usually manifests 2–4 h after meals with a spectrum of autonomic symptoms and neuroglycopenic symptoms, with severe episodes occasionally progressing to loss of consciousness or seizures [5, 6, 7]. Severe hypoglycemia can lead to substantial health complications that extend beyond immediate symptomatic episodes [8, 9]. This condition significantly impairs quality of life and has been associated with adverse cardiovascular outcomes, and the cognitive consequences of hypoglycemic episodes range from transient confusion to seizures [10, 11, 12]. The incidence of PBH varies widely in the literature, ranging from 2.6% to more than 30%, depending on the surgical technique, diagnostic criteria, and follow‐up duration [13, 14, 15, 16]. This variability underscores the challenge in identifying patients at risk.

The pathophysiology of PBH is complex and involves rapid gastric emptying, which leads to excessive glucose delivery to the small intestine, an exaggerated incretin response, and a postprandial insulin surge [17]. This hyperinsulinemic response is accentuated after RYGB; pathophysiologic processes have been documented after SG [6, 18]. Additional contributors include heightened β‐cell sensitivity, altered gut‐hormone profiles, reduced glucagon response, greater postoperative insulin sensitivity, and reduced insulin clearance [19, 20].

Despite these insights into PBH, patient‐level risk factors remain poorly defined across diverse surgical types and populations. Previous studies on PBH have primarily focused on hormonal mechanisms, often relying on limited sample sizes, homogeneous populations, or single‐center settings. Identifying preoperative risk factors for PBH is crucial for optimizing procedure selection and providing targeted preoperative counseling for high‐risk patients. To address this gap, we conducted a multicenter retrospective cohort study to examine clinical and demographic factors associated with PBH among patients undergoing RYGB and SG.

## 2 Materials and Methods

**FIGURE 1:**
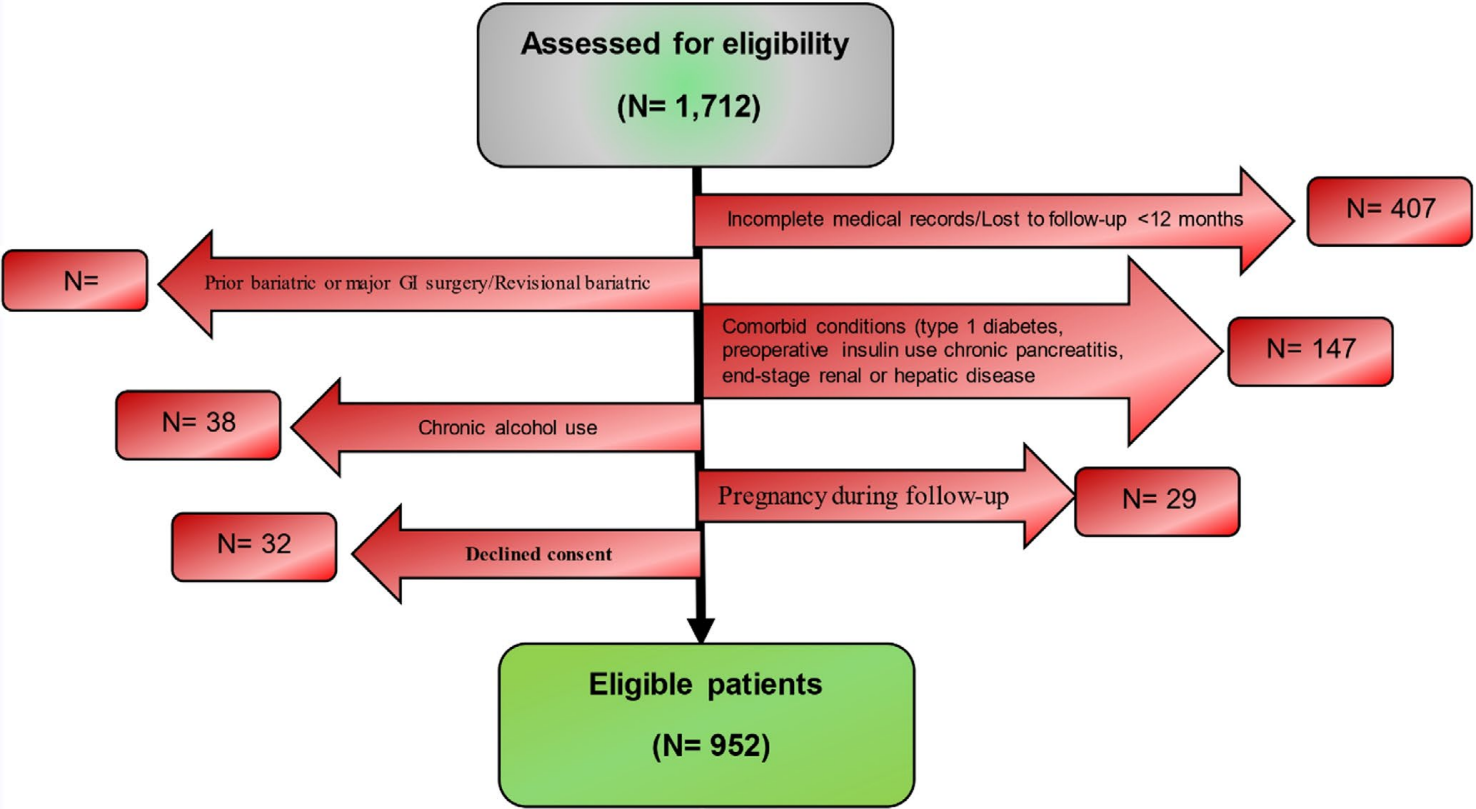
Participant flow diagram showing reasons for exclusion and numbers at each stage.

This retrospective cohort study was conducted at three affiliated medical centers of our institution from 2020 to 2024. We initially assessed the electronic health records (EHRs) of 1712 patients who underwent RYGB or SG surgery for inclusion in the study. Informed consent was obtained at the time of admission of patients for surgery. Ultimately, 952 patients met the study's inclusion criteria. Figure 1 presents the CONSORT‐style flow diagram of patient inclusion. Sampling was performed consecutively among the patients who underwent surgery. Patients were divided into two groups: those who developed PBH (*N* = 317) during follow‐up and those who did not (*N* = 635).

PBH was defined by Whipple's triad in a post‐bariatric patient: (i) clinician‐documented autonomic and/or neuroglycopenic symptoms; (ii) a temporally associated plasma ≤ 54 mg/dL (3.0 mmol/L); and symptom relief following carbohydrate [5]. Glucose values were obtained by capillary or venous plasma measurement at the time of symptoms (random/postprandial) during routine care or unscheduled presentations, when patients presented with symptoms in the clinic or emergency department. All patients had at least 12 months of follow‐up, and data were collected at 12, 15, and 18 months.

### 2.1 Inclusion and Exclusion Criteria

The inclusion criteria were patients aged 20–60 years, candidates for bariatric surgery (body mass index ≥ 35 kg/m^2^), and those with a minimum follow‐up of 12 months. Exclusion criteria included previous bariatric surgery or other major gastrointestinal surgery expected to alter anatomy or nutrient transit (including bowel resection, anti‐reflux surgery, and partial gastrectomy), revisional surgery, pregnancy during the follow‐up period, alcohol consumption, type 1 diabetes, diabetics taking insulin (preoperative insulin use), patients with preoperative vitamin B deficiency or malnutritionchronic pancreatitis, end‐stage renal or hepatic disease, and incomplete medical records.

### 2.2 Data Collection

Collected data included demographics (age, sex, education level), physical activity (minutes per week), comorbidities (diabetes, blood pressure, hypothyroidism, cardiovascular disease, obstructive sleep apnea, gastroesophageal reflux disease), smoking status, BMI, type of preoperative diabetes treatment (oral agents), and vitamin deficiencies (B1 or B12), diabetes duration, laboratory tests (HbA1c, cholesterol, HDL, LDL, triglycerides, fasting blood sugar [FBS]), fatty liver disease grade, and type of surgical procedure (SG vs. RYGB) were extracted by the researcher using a checklist. These variables were collected to assess their association with PBH. Vitamin B1 and B12 deficiency were defined preoperatively as serum levels below the local laboratory reference range within 3 months before surgery. Vitamin status data were obtained as part of the routine preoperative workup within 3 months before surgery.

### 2.3 Surgical Standardisation

All bariatric procedures were performed laparoscopically following a unified protocol across all centers. SG was carried out over a 32–40 French bougie, with gastric resection initiated 1–2 cm from the gastroesophageal junction to create a sleeve reservoir of approximately 75–150 mL. RYGB was performed with a small gastric pouch (~30 mL) and jejunojejunostomy reconstruction: the biliopancreatic limb was measured at ~50–75 cm from the ligament of Treitz, and the Roux (alimentary) limb at 100–150 cm. Mesenteric defects were routinely closed to prevent internal herniation. Leak testing of all anastomoses was performed intraoperatively. Postoperatively, patients followed a standardised diet progression and received routine follow‐up in nutrition and endocrinology. This uniform surgical approach across centers reduced variability in anatomic outcomes and enhanced the generalizability of our findings.

### 2.4 Postoperative Dietary Management and Patient Education

No uniform postoperative diet was mandated across centers. Each center followed its bariatric nutrition protocol under dietitian supervision, including: (i) staged advancement from clear liquids to puréed and soft textures, then to regular solids as tolerated; (ii) a protein‐first approach with restriction of rapidly absorbable carbohydrates and preference for low–glycemic index foods; small, frequent meals with separation of fluids from meals; and (iv) routine micronutrient supplementation per institutional standards (multivitamin/mineral plus thiamine and vitamin B12, with additional calcium/vitamin D and iron as clinically indicated). Patients with diabetes were evaluated by the endocrinology team for early deintensification of glucose‐lowering therapy, including insulin, to reduce hypoglycemia risk. All patients received counselling on recognition and initial management of PBH, including capillary glucose testing at symptom onset and instructions to seek care for severe or recurrent episodes.

### 2.5 Statistical Analysis

Data were analyzed using IBM SPSS Statistics 20.0 software. Quantitative variables were reported using means and standard deviations, and qualitative variables were reported using frequencies and percentages. The Shapiro–Wilk test was used to assess the normality of quantitative data. For continuous variables, the *t*‐test was used to analyze the parametric difference in means, and the Mann–Whitney *U* test was used in non‐parametric conditions. The chi‐square test (*χ* ^2^ test) was performed for categorical variables. Variables that had a *p*‐value < 0.15 in univariate analysis were entered into multivariate analysis using the backward method. Twelve months after surgery, multivariate logistic regression analysis was used to evaluate predictors of PBH. *p*‐values < 0.05 were considered statistically significant.

## 3 Results

Among 1009 patients, the mean age was 42.6 ± 8.3 years, and 778 (81.7%) were female. The educational level of the majority of the patients was a high school graduate or less. 449 (44.5%) patients had type 2 diabetes. The mean BMI of the patients was 38.1 ± 2.9. The mean duration of diabetes was 2.9 ± 1.1 years. Metformin, sulfonylureas, and sodium‐glucose cotransporter‐2 (SGLT2) inhibitors were the most commonly used antidiabetic medications. Mean HbA1c was 7.61% ± 1.20% in patients with diabetes versus 5.46% ± 0.32% in those without (*p* < 0.001). The mean FBS in diabetic and non‐diabetic patients was 146.1 ± 21.2 and 94 ± 8.1, respectively (*p*: 0.001). Baseline characteristics are summarized in Table 1. The cumulative incidence of PBH was 25.9% at 12 months, 29.3% at 15 months, and 35.4% at 18 months.

**TABLE 1:** Demographic and clinical characteristics of patients at baseline and in both groups with and without PBH at baseline.

Variable	All patients (*n* = 952)	Group		*p*
		With PBH (*n* = 317)	Without PBH (*n* = 635)	
Age at surgery (year)	42.6 ± 8.3	42.9 ± 8.4	41.6 ± 8.2	0.28
Sex				0.009
Male	174 (18.3%)	28 (8.8%)	146 (23%)	
Female	778 (81.7%)	289 (91.2%)	489 (77%)	
Education level				0.016
< high school graduate	334 (35.1%)	130 (41%)	198 (32.1%)	
High school graduate	341 (35.8%)	107 (33.8%)	240 (36.9%)	
> high school graduate	277 (29.1%)	80 (25.2%)	197 (31%)	
BMI (kg/m^2^)	38.1 ± 2.9	38.9 ± 2.7	37.3 ± 2.9	0.025
Smoking	275 (28.9%)	85 (26.8%)	190 (29.9%)	0.087
Comorbidities (Individual)				
Type 2 diabetes	424 (44.5%)	92 (29%)	332 (52.3%)	0.001
Hypothyroidism	133 (14%)	42 (13.2%)	91 (14.3%)	0.71
Hypertension	334 (35.1%)	105 (33.1%)	229 (36.1%)	0.29
Antidiabetic medications				0.001
Metformin	401 (42.1%)	104 (32.8%)	297 (46.8%)	
Sulfonylureas	387 (40.7%)	143 (45.1%)	244 (38.4%)	
SGLT2 inhibitors	164 (17.2%)	70 (22.1%)	94 (14.8%)	
Diabetic duration (year)	2.9 ± 1.1	1.81 ± 1.3	3.5 ± 0.98	0.004
Postoperative vitamin deficiency	441 (43.7%)	207 (58%)	234 (35.9%)	0.001
Laboratory tests (mean ± SD)				
HbA_1_c	6.3 ± 1.1	5.7 ± 1.1	7.32 ± 1.5	0.001
Cholesterol	215.2 ± 42.1	210.2 ± 40.1	222.1 ± 44.2	0.7
HDL	54.5 ± 12.5	52.2 ± 11.4	57.1 ± 13.2	0.56
LDL	128.9 ± 31.1	125.9 ± 30.2	130.2 ± 32.4	0.39
Triglycerides	175.2 ± 94.2	171.2 ± 93.1	179.3 ± 95.4	0.42
FBS (mg/dl)	131.1 ± 41.1	110.2 ± 35.2	168.2 ± 39.8	0.001
Fatty liver disease grading				0.081
None	426 (44.7%)	132 (41.8%)	294 (46.3%)	
1	239 (25.1%)	83 (26.2%)	156 (24.6%)	
2	189 (19.9%)	67 (21.1%)	122 (19.3%)	
3	98 (10.3%)	35 (11%)	63 (9.8%)	
Surgery type				0.006
SG	287 (30.1%)	60 (19%)	227 (35.7%)	
RYGB	665 (69.9%)	257 (81%)	408 (64.3%)	

### 3.1 Univariate Analysis

The prevalence of female patients was significantly higher in the PBH group than in the non‐PBH group (91.3% vs. 76.4%, *p* = 0.009). The proportion of illiterate patients or those with an education level below a high school diploma was significantly higher in the PBH group. The mean HbA1c and FBS levels in the PBH group were significantly lower than in the non‐PBH group. The mean duration of diabetes in the PBH and non‐PBH groups was 1.81 ± 1.3 and 3.5 ± 0.98 years, respectively (*p* = 0.004). The type of surgery was significantly associated with PBH, and the incidence of PBH was higher after RYGB than after SG. No significant differences were observed for other variables in the two groups (Table 1).

### 3.2 Multivariate Analysis

The results of multivariate analysis showed that female sex, education level below high school graduate, vitamin B1/B12 deficiency, and RYGB type surgery were significantly associated with an increased risk of PBH. While having type 2 diabetes, higher HbA1c levels, and longer duration of diabetes were significantly associated with a decreased risk of PBH (Table 2).

**TABLE 2:** Predictors of PBH incidence based on multivariate analysis.

Variable	OR	95% CI		*p*
		Lower	Upper	
Sex (female vs. male)	1.91	1.11	2.71	0.001
Education level (< high school graduate vs. > high school graduate)	1.61	1.1	2.11	0.009
Type 2 diabetes (yes vs. no)	0.75	0.55	0.96	0.006
Diabetic duration (year)	0.95	0.91	0.99	0.001
Vitamin deficiency (yes vs. no)	1.45	1.04	1.85	0.022
HbA_1_c (%)	0.97	0.95	0.99	0.001
Surgery type (RYGB vs. SG)	1.81	1.11	2.51	0.001

## 4 Discussion

This multicenter, retrospective observational cohort study demonstrated a steady increase in the cumulative incidence of PBH between 12 and 18 months post‐surgery. Cumulative incidence was 25.9% at 12 months, 29.3% at 15 months, and 35.4% at 18 months. In multivariable analyses, female sex, lower educational attainment, vitamin B1 and B12 deficiencies, and undergoing RYGB were associated with a higher risk of PBH. In contrast, preoperative type 2 diabetes, higher baseline HbA1c, and longer diabetes duration were associated with a lower risk. This pattern is consistent with prospective and multivariable analyses showing higher risk in women, after RYGB, and in those without pre‐existing diabetes [13, 14, 18, 21]. Fischer et al. [14] found that PBH occurred in up to one‐third of patients within 1 year after surgery. Salehi et al. [17] reviewed the pathophysiology of PBH, emphasising the role of rapid nutrient delivery and incretin‐mediated insulin secretion, especially following RYGB. Notably, the observed protection associated with higher preoperative HbA1c and longer diabetes duration is consistent with evidence that lower preoperative glycemia/HbA1c and preserved β‐cell function predispose to postprandial hypoglycemia after RYGB [13, 18].

The pathophysiology of PBH is complex, but it is hypothesized that exaggerated postprandial insulin responses driven by altered gut hormone dynamics are a primary factor in this condition [22]. Following RYGB, rapid transit of carbohydrates to the distal small intestine leads to significant glucose elevations, followed by rapid declines [17]. Research indicates that patients who have undergone RYGB with PBH demonstrate significantly elevated postprandial GLP‐1 levels and marked insulin surges compared to those who do not experience hypoglycemia [5, 7]. In a cohort of individuals with obesity and type 2 diabetes undergoing RYGB, higher 30‐ and 60‐min insulin concentrations during an Oral Glucose Tolerance Test (OGTT) at 6 months predicted PBH at 12 months, whereas glucagon levels and a calculated β‐cell area index were not associated with PBH risk [23]. These findings suggest that greater postprandial β‐cell responsiveness, rather than glucagon tone or surrogate measures of β‐cell mass, contributes to susceptibility in a subset with preserved function [5]. However, Lobato et al. [24] found that this relationship may not always exhibit direct correlation and suggested the involvement of additional complex regulatory mechanisms.

Early reports described diffuse islet hyperplasia (nesidioblastosis) after RYGB and led to distal or subtotal pancreatectomy for refractory PBH; however, the benefit was inconsistent, relapse was common, and morbidity was appreciable [25]. Contemporary guidance discourages pancreatectomy for PBH, except in rare cases of focal insulin‐secreting lesions, and instead favors dietary therapy with stepwise pharmacological management. Compared with SG, RYGB is associated with greater glycemic variability and more hypoglycemia on CGM, although SG also accelerates gastric emptying and can contribute to variability [26, 27]. The observations of Nilsen et al. [26] support our findings that glycemic variability and hypoglycemia are greater after RYGB than SG. Studies by Johari et al. [27] and Wickremasinghe et al. [28] confirmed that SG accelerates gastric emptying, which can contribute to glycemic variability.

In our cohort, lower educational attainment and vitamin B1/B12 deficiencies were associated with higher odds of PBH, highlighting the role of broader social and nutritional determinants. Because these are observational data, these findings should be interpreted as associations rather than causal effects and may, in part, reflect differences in dietary adherence, supplement compliance, and postoperative follow‐up intensity. Thiamine is an essential cofactor for enzymes involved in carbohydrate oxidation, and deficiency may impair postprandial glucose utilisation. Vitamin B12 deficiency has been associated with autonomic dysfunction, potentially attenuating adrenergic counter‐regulation and diminishing symptom awareness during hypoglycemia [29, 30]. However, current evidence does not establish preoperative B‐vitamin deficiency as a predictor of PBH. This association may represent overall baseline nutritional quality rather than causation. This association may reflect baseline nutritional status rather than a causal relationship. In the absence of detailed data on dietary intake and adherence to supplementation, residual confounding cannot be ruled out. Lower education level may indicate reduced health literacy or socioeconomic challenges, which can impact dietary adherence, follow‐up care, and the ability to recognise symptoms [31, 32]. Wright et al. [31] and Mahoney et al. [32] emphasised that lower health literacy is associated with worse outcomes after surgery. Given the frequency and morbidity of PBH, patient education and confirmation of hypoglycemia at the time of symptoms are essential. Routine care should include structured dietary counselling on meal composition, with a focus on avoiding simple sugars and early de‐escalation of glucose‐lowering therapy. Capillary glucose measurement during symptomatic episodes facilitates diagnosis and management; in patients with severe or recurrent events, continuous glucose monitoring (CGM) may be considered.

Consistent with previous studies [14, 33], we found that female patients were more likely than male patients to experience PBH. Observational studies indicate that women exhibit higher insulin sensitivity than men, independent of age and BMI [34, 35]. Experimental data suggest that this advantage reflects skeletal‐muscle characteristics and sex‐steroid signaling that enhance insulin action [36, 37, 38]. In premenopausal women, cyclical fluctuations in oestrogen and progesterone are associated with enhanced insulin sensitivity and heightened incretin‐mediated β‐cell responsiveness [39, 40]. Further mechanistic investigation of insulin, glucagon, and GLP‐1 signaling pathways is warranted to inform the development of targeted therapies. Studies conducted by Loh NY et al. [41] and Mauvais‐Jarvis et al. [42] have underscored the role of sex hormones in glucose metabolism and insulin sensitivity, which may explain these differences. Additionally, the higher participation of women in most studies may contribute to this trend.

Patients with preoperative type 2 diabetes, particularly those with higher baseline HbA1c and longer diabetes duration, showed a lower risk of PBH. In advanced diabetes, progressive β‐cell dysfunction limits postprandial hyperinsulinemia, providing some protection against PBH [18, 43]. Conversely, patients requiring insulin preoperatively may have partially preserved β‐cell function but significant insulin resistance; after surgery, the rapid improvement in insulin sensitivity, combined with enhanced GLP‐1–mediated insulin secretion, may lead to relative insulin excess and hypoglycemia if exogenous insulin dosing is not promptly adjusted [42, 44].

### 4.1 Strengths and Limitations

However, our study had limitations that should be noted. The retrospective design carries the risk of selection and information bias. Diagnosis of PBH was based on Whipple's triad using EHR documentation of symptom‐timed plasma glucose values, rather than CGM or standardised provocative testing, such as OGTT and mixed‐meal tests. Some variables, such as education level and supplement use, were self‐reported and susceptible to misclassification. We did not systematically capture risk factors previously linked to PBH, such as prior cholecystectomy and specific medication classes, as well as a specific classification of vitamin B deficiencies, which may have resulted in residual confounding. Prospective studies incorporating continuous glucose monitoring and standardised testing are warranted to validate these findings and further explore preventive strategies. Despite these limitations, our study offers valuable insights into which patients are at the greatest risk of PBH and highlights the importance of postoperative management in mitigating this risk.

Strengths of this study include its multicenter design, large sample size, and comprehensive analysis of demographic, clinical, and surgical predictors.

## 5 Conclusion

PBH is a significant postoperative complication. Female sex, lower education, preoperative vitamin B1/B12 deficiency, and undergoing RYGB were independently associated with a higher risk of PBH. In contrast, type 2 diabetes, higher baseline HbA1c, and longer diabetes duration were protective factors. Identifying these predictors can help guide monitoring and postoperative management to reduce the incidence of PBH and improve outcomes.

## Author Contributions

Conception and design: A.T. and M.B. and M.S. Analysis and interpretation: M.B. and B.G. Data collection: A.A.G., A.M., D.Y., and A.R. Participation in drafting or revising the article: M.B., M.S., and M.H.

## Ethics Statement

This retrospective chart‐review study was approved by the Ethics Committee of Iran University of Medical Sciences. The study was conducted at centers affiliated with Iran University of Medical Sciences and Shahid Beheshti University. All procedures conformed to the principles of the Declaration of Helsinki.

## Consent

Informed consent was obtained at the time of admission of patients for surgery.

## Conflicts of Interest

The authors declare no conflicts of interest.

## Data Availability

The data that support the findings of this study are available from the corresponding author upon reasonable request.
